# ACUDIN – ACUpuncture and laser acupuncture for treatment of DIabetic peripheral Neuropathy: a randomized, placebo-controlled, partially double-blinded trial

**DOI:** 10.1186/s12883-018-1037-0

**Published:** 2018-04-13

**Authors:** Gesa Meyer-Hamme, Thomas Friedemann, Henry Johannes Greten, Rosemarie Plaetke, Christian Gerloff, Sven Schroeder

**Affiliations:** 1 HanseMerkur Center for Traditional Chinese Medicine at the University Medical Center Hamburg-Eppendorf, Martinistrasse 52, House O55, 20246 Hamburg, Germany; 2 Heidelberg School of Chinese Medicine, Karlsruher Str. 12, 69126 Heidelberg, Germany; 3 Department of Neurophysiology, Instituto di Ciencias Biomedicas Abel Salazar, University of Porto, Rua de Jorge Viterbo Ferreira n. 228, 4050, –313 Porto, Portugal; 4 Department of Medical Biometry and Epidemiology, University Medical Center Hamburg-Eppendorf, Martinistrasse 52, 20246 Hamburg, Germany; 5 Department of Neurology, University Medical Center Hamburg-Eppendorf, Martinistrasse 52, 20246 Hamburg, Germany

**Keywords:** Diabetic peripheral neuropathy, Acupuncture, Laser acupuncture, Placebo-control, Randomized controlled trial, Nerve conduction studies, Neurography

## Abstract

**Background:**

Diabetic peripheral neuropathy (DPN) is the most common complication of diabetes mellitus with significant clinical sequelae that can affect a patient’s quality of life. Metabolic and microvascular factors are responsible for nerve damage, causing loss of nerve function, numbness, painful sensory symptoms, and muscle weakness. Therapy is limited to anti-convulsant or anti-depressant drugs for neuropathic pain and paresthesia. However, reduced sensation, balance and gait problems are insufficiently covered by this treatment. Previous data suggests that acupuncture, which has been in use in Traditional Chinese Medicine for many years, may potentially complement the treatment options for peripheral neuropathy. Nevertheless, more objective data on clinical outcome is necessary to generally recommend acupuncture to the public.

**Methods:**

We developed a study design for a prospective, randomized (RCT), placebo-controlled, partially double-blinded trial for investigating the effect of acupuncture on DPN as determined by nerve conduction studies (NCS) with the sural sensory nerve action potential amplitude as the primary outcome. The sural sensory nerve conduction velocity, tibial motor nerve action potential amplitude, tibial motor nerve conduction velocity, the neuropathy deficit score, neuropathy symptom score, and numeric rating scale questionnaires are defined as secondary outcomes. One hundred and eighty patients with type 2 diabetes mellitus will be randomized into three groups (needle acupuncture, verum laser acupuncture, and placebo laser acupuncture). We hypothesize that needle and laser acupuncture have beneficial effects on electrophysiological parameters and clinical and subjective symptoms in relation to DPN in comparison with placebo.

**Discussion:**

The ACUDIN trial aims at investigating whether classical needle acupuncture and/or laser acupuncture are efficacious in the treatment of DPN. For the purpose of an objective parameter, NCS were chosen as outcome measures. Acupuncture treatment may potentially improve patients’ quality of life and reduce the socio-economic burden caused by DPN.

**Trial registration:**

German Clinical Trial Register (DRKS), No. DRKS00008562, trial search portal of the WHO (http://apps.who.int/trialsearch/).

## Background

Diabetic peripheral neuropathy (DPN) is the most common complication of diabetes mellitus with significant clinical sequelae and impact on patients’ quality of life [1, 2]. DPN manifests itself on the toes and progresses in a stocking distribution [3]. Nerve damage is related to hyperglycemia. However, various other mechanisms play a role in the pathogenesis of DPN [3]. These include elevated polyol pathway activity, advanced glycation end products (AGEs), oxidative stress, growth factors, impaired insulin/C-peptide action, and elevated protein kinase C activity. These may directly affect neuronal tissues as well as vascular structures, thus compromising nerve vascular supply [4, 5].

Clinical manifestations include paresthesia, burning sensations, and neuropathic pain as well as negative symptoms like hypesthesia, hypalgesia, and pallhypesthesia. These may contribute to balance problems and unsteady gait, leading to falls [6] and an increased risk of bone fractures and hospitalization [7]. Motor symptoms, for example muscle spasm and weakness occur less frequently [3]. DPN is associated with an increased risk of ulceration and amputation of the lower extremities as well as increased healthcare costs [8–10]. Nerve damage can occur on the myelin sheath as well as at the axonal level [11]. Differentiation is achieved by nerve conduction studies (NCS) [12]. Whilst neuropathic pain and paresthesia can be palliated by anti-convulsants, tricyclic antidepressant drugs or serotonin-noradrenalin re-uptake inhibitors [13], pharmacologic management of decreased sensation is generally ineffective, thus forming a gap in treatment strategies.

During the last decades, acupuncture has become an empirical complementary treatment option for DPN. It is recommended by the World Health Organisation [14] and the National Institute of Health [15] but treatment effectiveness is still under debate. Reviews concerning acupuncture for peripheral neuropathy (PN) have found that, despite the majority of studies reporting positive results, a reliable statement of effectiveness is not possible due to methodological limitations [16–22]. Randomized controlled, blinded clinical trials of adequate statistical power and design are still pending.

Whilst acupuncture concepts using proximal or systemic acupuncture points on upper and lower extremities failed to show efficacy [23], our pilot studies using the selection of local and distal points described here showed promising results for the treatment of DPN, chemotherapy-induced PN and PN of unknown cause(s). These studies have verified the improvement that can be found in subjective scales by means of NCS parameters [24–27]. Acupuncture has been shown to increase blood perfusion towards the periphery of the limbs following needle insertion [28]. The results of our pilot studies were potentially related to the acupuncture effect on the blood flow through vasa nervorum and dependent capillary beds supplying the neurons [26]. These findings are encouraging for setting up the framework of a clinical study of adequate sample size using strict methodological standards.

### Definition of adequate controls for acupuncture

The development of appropriate designs for clinical acupuncture trials remains a methodological challenge. A major problem is the definition of placebo controls and blinding procedures, as the insertion of acupuncture needles is usually perceptible and visible.

Double-blinding of needle acupuncture has been achieved for immediate effects using invasive sham acupuncture concepts as control [29, 30]. However, this approach is problematic for long-term trials because of a possible overlap of specific as well as unspecific physiological and placebo effects. Sham acupuncture, at irrelevant or non-acupuncture points, was found to promote physiological stimuli due to an unspecific endorphin release in a range of 33–50%, exceeding the effect of a suggestive placebo therapy [31]. Hence, treatment with invasive sham acupuncture is not an inert placebo but an active treatment of unknown activity [32].

Accordingly, no reliable blinding methodology for needle acupuncture has been achieved so far for time spans of 10 weeks’ intervention. Differences in verum and placebo treatment patterns could not be accurately masked from the practitioner over time, creating the risk of bias [33].

Even shallow needling showed both specific and unspecific effects [34–36], evoking physiological responses similar to classical needle acupuncture and distorting the results [37].

To overcome these obstacles we include Laserneedle® acupuncture with multichannel red laser light into the ACUDIN study design. Laser needles are not inserted into the skin but merely placed on the surface of the acupuncture points; however, the therapeutic effects are of a similar dimension to those evoked by manual needle acupuncture [38]. The initial effect of laser acupuncture is mediated by the impact of ATP release from cutaneous mast cells [39, 40], which is considered comparable to the mechanical effect of needle acupuncture [41, 42]. Patients do not feel the activation of the laser needles as the radiation intensity is optimized for this purpose [43] and they cannot distinguish between verum and placebo laser [44]. Laser acupuncture is considered an appropriate means of control for acupuncture trials for evaluating the effect of needling per se [44].

## Methods/design

### Trial design

The aim of the ACUDIN trial is to examine if acupuncture and laser acupuncture have beneficial effects on NCS parameters, clinical scores and patient’s complaints in DPN as evaluated by standardized tests. A prospective, randomized, placebo-controlled, partially double-blinded, three-armed study design has been applied to the ACUDIN trial to compare the effects of classical needle acupuncture and laser acupuncture with those of placebo laser acupuncture.

**Fig. 1:**
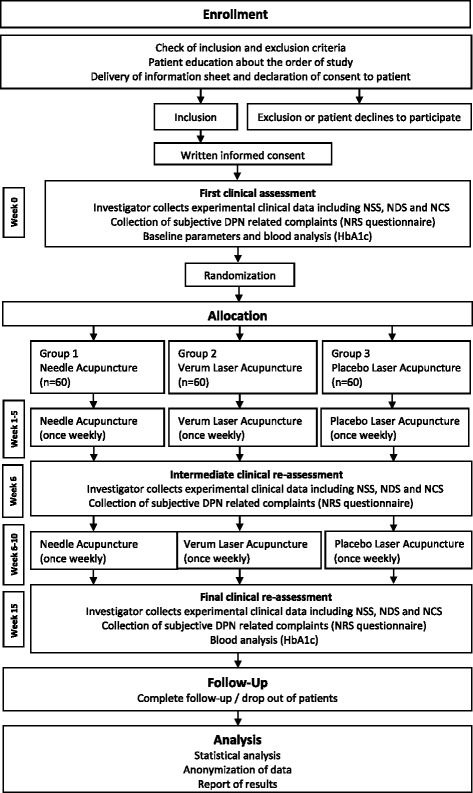
Depicts the ACUDIN trial protocol

Patients will receive 10 treatments over a period of 10 weeks. Each session will last 20 min. Neurological assessment, including NCS, is performed at baseline, week 6 and week 15 (Fig. 1).

### Trial personnel

A total of four persons are involved in the ACUDIN trial procedure. The treatment is performed by the following health professionals: (i) experienced practitioners who are members of a German Physicians Society for Acupuncture. They have completed a standardized training course, undertaken formal accreditation by examination and a period of supervised medical experience required for administering the acupuncture interventions and were trained by Laserneedle® specialists for the implementation of multichannel laser acupuncture; (ii) two study nurses are involved in the treatment procedure as described below; and (iii) experienced neurologists, not involved in further study procedures, perform neurological assessments including NCS.

### Study population

A total of 180 patients with DPN due to diabetes mellitus type 2 will be recruited from regional medical clinics, via the homepage of the operating institute, media reports, and advertisements.

### Inclusion criteria

Participants who meet the following conditions will be included:

- Male or female aged > 18 years
- Confirmed diagnosis of diabetes mellitus type 2 (i.e., HbA_1c_ ≥ c6,5% (48 mmol/mol)

[45] and/or on diabetes medication for more than 1 year)

- Stable levels of HbA_1c_ during the last 6 months (i.e., deviation < 1% (11 mmol/mol))
- Clinically confirmed diagnosis of DPN
- Pathologic results in NCS (i.e., sural SNAP < 10 μV, sural NLG < 42 m/s, tibial MNAP < 8 mV, tibial MNLG < 40 m/s) [46]
- Naive to laser acupuncture
- No prior acupuncture treatment for peripheral neuropathy

### Exclusion criteria

Participants who present one or more of the following conditions are excluded:

- PN caused by conditions other than diabetes (e.g., alcohol abuse, chemotherapy, hereditary causes, chronic inflammatory or idiopathic PN, and others)
- History of epilepsy
- Coagulopathy or use of anticoagulants with bleeding time > 3 min, prothrombin time < 40%, platelet count < 50.000/μl, or PTT > 50 s
- Bacterial infection or other skin diseases at the lower extremities that impede acupuncture treatment
- Bone fracture of the lower extremities during the last 3 months
- Use of acupuncture during the last 3 months
- Opiate, analgesic, or drug abuse
- Psychiatric illnesses other than mild depression
- Incapacity in giving informed consent or in following the study instructions due to language disturbances, serious cognitive deficits, or lack of time
- Pregnant or breast-feeding women
- Current participation in other clinical studies

### Randomization procedure

Recruited patients are randomized in a ratio of 1: 1: 1 into three parallel treatment groups of 60 participants: (i) classical needle acupuncture, (ii) verum laser acupuncture, and (iii) placebo laser acupuncture. Randomization is prepared beforehand with 180 identical closed envelopes, each.

containing one of the three possible group allocations and a randomly generated four-number pseudonym code for further data processing. Randomization is performed by envelope lottery immediately prior to the first treatment session by study nurse 1. Patients and practitioners are only informed about the patients’ allocation to either needle or laser treatment. However, patients do not know whether they will receive a true treatment or placebo. Group assignment will not be exposed until the final data analysis report is completed.

## Blinding

A single-blind design is performed for needle acupuncture because of the difficulties of practitioner blinding for the needling procedure as specified above. A double-blind design is performed for laser acupuncture. Caregiver blinding for laser acupuncture is achieved by dividing the treatment into three steps.

i. practitioner fixes the laser needles without activating them;
ii. study nurse 1 operates the laser device according to the randomization (verum or placebo);
iii. study nurse 2 removes the laser needles after treatment.

Practitioners and study nurses do not communicate concerning the patients’ group allocation. Further, the investigator performing the neurological assessment and NCS does not know about the type of treatment given to the patient.

Regarding patients and the blinding procedure: the laser device is placed behind a folding screen so patients cannot see it. Patients wear laser protective glasses which make it impossible to perceive the laser light. They are informed that the laser device would emit visible and/or invisible laser beams and that the protection of the eyes is fundamental in any case because of the safety precautions as required by law. The laser needles are covered with a blanket. Acoustic functions of the laser device are deactivated prior to the study, so there is no difference between verum and placebo activation. This protocol will not be published in advance so patients cannot draw any conclusions concerning treatment allocations. Study participation is allowed only once.

For data transfer and processing, pseudonym codes are used in order to mask patient identity and treatment groups from outcome assessors and statisticians. The data is only matched after completion of statistical analysis.

## ACUDIN acupuncture protocol

A total of 20 acupuncture points have been selected for the ACUDIN trial. All treatment sessions of needle and laser acupuncture are performed using the following point combination:

The 4 Bafeng points on both feet (Ex-LE-10) [47]. Needles are inserted to a depth of 3 to 5 mm and left in place without stimulation for 20 min. Laser needles are fixed with perforated plaster.

**Fig. 2:**
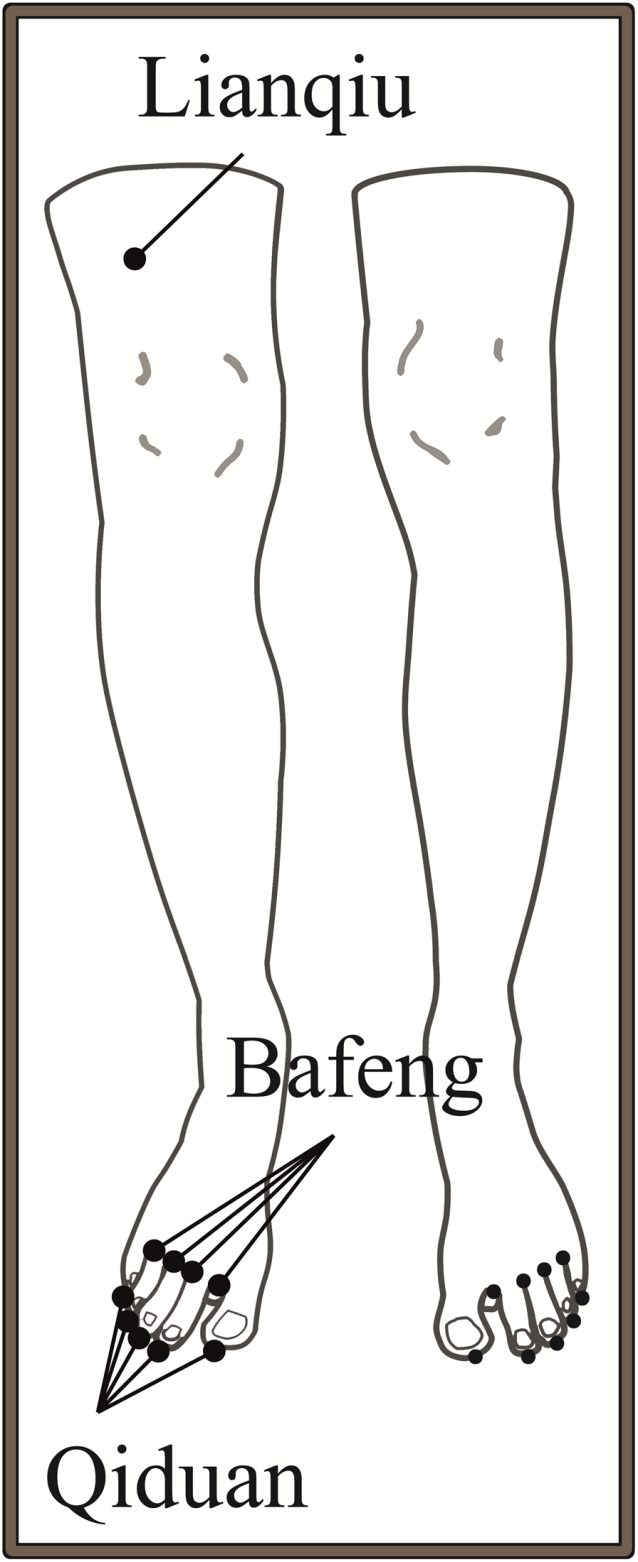
Illustrates the ACUDIN acupuncture point selection

The 5 Qiduan points on both feet (Ex-LE-12) [47]. Needles are inserted to a depth of 1,5 to 2 mm and left in place without stimulation for 20 min. Laser needles are fixed with perforated plaster (Fig. 2).

The point Lianqiu on both legs (ST-34) [47]. Needles are inserted to a depth of 0,5 to 2.5 cm (depending on the diameter of the thigh) and left in place without stimulation for 20 min. Laser needles are fixed with perforated plaster.

Sterile single-use stainless steel needles size 0,2 × 15 mm are used for Qiduan and Bafeng points, respectively 0,3 × 30 mm for Lianqiu, both manufactured by Wujiang City Cloud & Dragon Medical Devise Co. Ltd, China.

### Laser acupuncture device

Laser acupuncture is performed with Laserneedle® device (European patent PCT/DE 102006008774.7), a multichannel class 3B system which allows the simultaneous stimulation of individual point combinations through semiconductor laser diodes. Flexible optical light fibers conduct the laser light without loss, providing a high optical density at the distal end [40]. Wavelengths of 685 nm (red light) are emitted in continuous mode. Each channel has an optical power of 35 mW. Power density is 2.3 kJ/cm^2^ per channel. The beam diameter is 500 μm and beam divergence at the end of the laser needle is 9.5. Laser needles are placed at an angle of 90° directly on the skin, thus radiation reflection is minimized [43]. Laser needles are plugged in silicon adapters which are fixed with perforated plaster to the skin, so they stay in place without requiring the practitioner to stay with the patient. Temperature monitoring is not required because of extremely low heat development due to the weak absorption coefficient at 685 nm [48]. Previous investigations revealed no unintended side-effects of Laserneedle® treatment [40, 48]. The use of laser protection glasses is obligatory for both study nurse and patient. Two Laserneedle® machines with 10 laser needles each are used for the ACUDIN trial.

## Procedures

### Verum needle acupuncture

The treatment is applied according to the protocol with the patient in the supine position. The practitioner inserts the acupuncture needles after local disinfection of the skin. Needles are left in place for 20 min. They are carefully removed and disposed into a bedside sharps container at the completion of treatment by study nurse 2.

### Verum laser acupuncture

The treatment is applied according to the protocol with the patient in supine position. The practitioner fixes the laser needles with perforated plaster after local disinfection of the skin. The patient’s legs and feet are covered and he/she wears laser protection glasses. The practitioner then leaves the room. Study nurse 1 enters the room and activates the laser emission by pressing at the soundless touch screen of the laser device according to the randomization schedule. Laser emission stops automatically after 20 min. Laser needles are removed and cleaned after use by study nurse 2.

### Placebo laser acupuncture

Placebo laser acupuncture is performed as described for verum laser acupuncture with slight modifications. Study nurse 1 provides the same steps as for the verum treatment but without activating the laser, pressing an invalid point at the soundless touch screen of the laser device. Therefore, from the patient’s perspective, the procedure is identical to that used in the verum laser group.

### Nerve conduction studies

The ACUDIN trial outcome measures are based on NCS parameters that are both objective and reproducible. These measures also provide quantitative data according to the Guideline for Diabetic Neuropathy [49, 50]. NCS are performed on both legs with a Neuropack-Sigma, MEB-9400, EMG/NCV/EP-System (Nihon-Khoden, Japan). Sural SNAP and tibial MNAP are measured on the first negative peak. All studies on NCS are done at room temperature (23 ± 1 °C). Sural NCS are done with standard orthodromic needle recording methods [51]. Tibial NCS are done with standard orthodromic surface electrode recording methods [51]. Skin temperatures are measured, and values of NCV are adjusted for the effect of temperature.

### Clinical examination

Clinical examinations include sensory qualities such as the perception of pain and temperature, pallesthesia and two-point discrimination, patellar and Achilles reflexes, and gait qualities. The neuropathy deficit score (NDS) [52] and the neuropathy symptom score (NSS) [52] are part of the clinical assessment. Both are validated instruments for diagnosis and progress of DPN according to the Guideline for Diabetic Neuropathy [49].

### Questionnaires to collect participants’ opinions

Common DPN-related symptoms will be recorded weekly by questionnaires using 11-point numeric rating scales (NRS) with the terminal descriptors “no complaint” and “worst complaint possible”. Whilst NRS are well validated for painful conditions [53], our questionnaires assess not only neuropathic pain but also tingling, burning pain, feeling of heat, feeling of cold, cramps, numbness, gait impairment, impairment of daily activities, sleep disturbances, and complaint frequency.

Table 1 summarizes the plan of data collection.

**Table 1:** Baseline and outcome parameters

	Baseline parameter	Blood parameter	Neurological assessment	NCS^f^ Parameter	Subjective Score
Week 0	Age Gender Weight Size Comorbidity Medication Duration of disease (Diabetes and DPN)	HbA_1c_^a^	Clinical examination NSS NDS^c^	Bilateral amplitudes and NCV^b^of nn. surales and tibiales	NRS^d^
Week 6			Clinical examination NSS^e^ NDS	Bilateral amplitudes and NCV of nn. surales and tibiales	NRS
Week 15	Change of medication	HbA_1c_	Clinical examination NSS NDS	Bilateral amplitudes and NCV of nn. surales and tibiales	NRS Adverse events
Weekly (treatment 1–10)	Change of medication				NRS Adverse events

^a^HbA1c= glycated hemoglobin; ^b^NCV = nerve conduction velocity; ^c^NDS = neuropathy deficit score; ^d^NRS = numeric rating scale; ^e^NSS = neuropathy symptom score; ^f^NCS = nerve conduction studies

## Outcome measures

### Primary endpoint

The primary endpoint is the difference of the 15-week measurement minus baseline of the sural sensory nerve action potential amplitude (sural SNAP).

### Key secondary endpoints

Secondary endpoints are the differences of the 15-week measurement minus baseline of the remaining NCS measurements:

the sural sensory nerve conduction velocity (sural SNCV),

tibial motor nerve action potential amplitude (tibial MNAP),

tibial motor nerve conduction velocity (tibial MNCV).

Additional secondary endpoints will be obtained from the questionnaires:

The differences in the 15-week measurement minus baseline of NDS, NSS, and NRS scores.

## Statistical analysis

Major hypotheses tested are:

H0:

The differences of the 15-week measurements minus baseline of sural SNAP in the two groups with classical needle acupuncture or laser acupuncture are the same or smaller as in the control group.

vs.

H1:

The differences of the 15-week measurement minus baseline of sural SNAP in the groups with classical needle acupuncture or laser acupuncture are larger than in the control group.

In addition, for the two types of acupuncture treatment, two-sided hypotheses will be tested.

### Data analysis

Hypotheses will be tested by using one-way analysis of variance (ANOVA), followed by Tukey post-hoc tests comparing the three groups pairwise.

A reasonable approximation to a normal distribution, according to the central limit theorem, can be assumed with the calculated sample size, allowing the use of parametric analysis. Deviations of the normal distribution will be tested and appropriate transformations will be performed. Homogeneity of variance will be tested with the Levene’s test.

The primary analysis will be performed with the intent-to-treat (ITT) population. Missing data will be imputed by the next-observation-carried-backwards option for initial values and last-observation-carried-forwards option for closing values. Legs with missing initial and closing data for the primary endpoint will be deleted from the analysis. The average values of both legs will be evaluated for patients with bilateral assessable measurements. For unilateral results, only the evaluable leg will be included in the analysis. The comparison between verum needle acupuncture and placebo laser acupuncture will be undertaken for validating the overall treatment effect of acupuncture. Verum laser acupuncture and placebo laser acupuncture will be compared for validating the specific treatment effect. The comparison of needle acupuncture to verum laser acupuncture will indicate the non-specific effect of needle acupuncture. Remaining NCS outcomes will be analyzed as described for the primary outcome. Multivariate analyses of the clinical secondary outcomes (NDS, NSS, and NRS) and their correlation to NCS parameters will be performed by linear regression models.

Baseline data and clinical characteristics will be presented as mean values to assess the baseline comparability of the intervention groups. Those patients who report changes of pain medication during the study will be excluded from data analysis relating to subjective pain scales. Statistical analysis will be done with the software SPSS (IBM SPSS Statistics 22) and OriginPro 9.

### Sample size calculation

An average difference of 1 μV was considered functionally relevant for sural SNAP [54]. On the basis of a pilot study [24], a pooled standard deviation of 1.83 μV was calculated. By assuming that this standard deviation represents the best estimate of the actual standard deviation for each of the three groups in a regular three-arm parallel RCT design with complete follow-up, 54 patients per arm are required to detect the above difference for sural SNAP with an alpha of 0.05 and a power of 0.8. The assumption of a conservative 10% drop-out rate for patients randomized to any of the three arms increases the sample size to 60 patient per arm or 180 patients in total.

Power evaluations were performed with statistical analysis system (SAS) 9.2 for the above described statistical analysis (one-way ANOVA) and followed by post-hoc testing for the primary outcome.

### Adverse events

Any adverse event and unexpected and unintended responses to the treatment reported by the patients are recorded weekly with the NRS questionnaire. A causal relationship between treatment and adverse events will be evaluated using a six-grade-scale with the terminal descriptors 1 = definitely related and 6 = unknown. Possible adverse events related to acupuncture are hematoma, pain, nerve irritation, infection, and tiredness. An accidental irradiation of the eyes is regarded as the only relevant adverse event related to Laserneedle® treatment [40].

### Timelines

Recruitment started in August 2011. Data collection started in January 2012. The ACUDIN trial is due to finish in 2017.

## Discussion

DPN is a common complication of diabetes mellitus [1, 2]. Conventional medicine offers symptomatic pharmaceutical treatment for DPN masking neuropathic pain and paresthesia [13]. Unfortunately, these treatments are not always effective [3] and patients have to cope with medication side-effects [55]. Furthermore, there is no treatment for sensory loss, unsteadiness, and gait-impairment, causing a gap in medical care for diabetic patients and giving reason for extending the range of treatment options.

Acupuncture has been in use for decades for the treatment of DPN and positive results have been reported on the basis of empirical knowledge and a number of pilot studies [16–22]. However, the effectiveness of acupuncture is still under debate because of the lack of high-quality clinical research.

The peripheral nervous system has the ability of regeneration in relation to repair mechanisms and in the phenomena of spreading and sprouting [56]. In DPN, morphologic peripheral nerve regeneration is impaired and inadequate nerve regeneration contributes to the pathophysiologic mechanism of DPN [57]. However, the findings of our pilot study mention a possible improvement of NCS parameters following acupuncture treatment in DPN, indicating a certain amount of neural repair [25, 26]. Parallel to improved NCS, patients have experienced subjective improvement after acupuncture treatment compared to no specific treatment, but experienced the best medical care where positive and negative DPN-related symptoms were concerned [25]. The acupuncture protocol of the pilot studies using distal and local points on the lower extremities is taken as the basis for the ACUDIN study design because other concepts failed to show efficacy [23].

This paper presents the protocol for the prospective, randomized, placebo-controlled, partially double-blinded, three-armed clinical ACUDIN trial for testing the hypothesis that classical needle acupuncture and laser acupuncture improve objective NCS parameters, as well as clinical and subjective symptoms in DPN compared to placebo treatment. NCS has rarely been used as an objective parameter in acupuncture research [24–26, 58–62]. The ACUDIN trial is the first study using NCS as an evaluation method of acupuncture treatment of DPN of the lower extremities in the form of a randomized, partially double-blinded design type.

DPN may have a strong negative impact on patients’ quality of life [13]. However, healthcare systems can encompass increased use of resources and costs which may escalate according to the severity of the disease [8–10]. Acupuncture treatment can offer a cost-effective treatment option in comparison with conventional medicine [63, 64].

The findings of the ACUDIN trial may establish the value of complementary acupuncture treatment for the potential improvement of peripheral nerve function and clinical symptoms; in addition, the findings may also lead to a reduction of the socio-economic burdens caused by DPN.

## Abbreviations

ACUDIN: ACUpuncture and Laser Acupuncture for treatment of DIabetic peripheral Neuropathy
ANOVA: Analysis of Variance
DPN: Diabetic Peripheral Neuropathy
HbA_1c_: Glycated Hemoglobin
HMC: HanseMerkur Center for Traditional Chinese Medicine at the University Medical Center Hamburg-Eppendorf, Germany
MNAP: Motor Nerve Action Potential
MNCV: Motor Nerve Conduction Velocity
NCS: Nerve Conduction Study
NCV: Nerve Conduction Velocity
NDS: Neuropathy DeficitScore
NRS: Numeric Rating Scale
NSS: Neuropathy Symptom Score
PN: Peripheral Neuropathy
RCT: Randomized Controlled Trial
SNAP: Sensory Nerve Action Potential
SNCV: Sensory Nerve Conduction Velocity
UKE: University Medical Center Hamburg-Eppendorf
WHO: World Health Organisation
